# From Recovery to Enhancement: Pressure-Gradient-Driven Crack Repair of Particulate-Reinforced Polymer Composites

**DOI:** 10.3390/polym18121485

**Published:** 2026-06-13

**Authors:** Shengnan Wang, Xinqiao Zhu, Wei Tang, Maoping Wen, Lingang Lan, Xin Tian, Hongwei Yuan

**Affiliations:** 1 National Key Laboratory of Chemical Explosion Safety, Institute of Chemical Materials, China Academy of Engineering Physics, Mianyang 621999, China; shengnan@caep.cn (S.W.); tangwei@caep.cn (W.T.); wenmp@caep.cn (M.W.); lanlg@caep.cn (L.L.); tianx87@caep.cn (X.T.); 2 Institute of Nuclear Physics and Chemistry, China Academy of Engineering Physics, Mianyang 621999, China; zhuxinqiao@zju.edu.cn; 3 Graduate School of China Academy of Engineering Physics, Beijing 100193, China

**Keywords:** particulate-reinforced polymer composites, crack repair, pressure-gradient-driven infiltration, mechanical enhancement, strain localization, interfacial strength

## Abstract

Particulate-reinforced polymer composites (PRPCs) are susceptible to cracking under tensile loading, severely limiting their service life. Here, we propose a pressure-gradient-driven infiltration method that rapidly repairs narrow (<10 μm) cracks in a highly filled PRPC (95 wt.% BaSO_4_/5 wt.% fluororubber). Microstructural evidence confirms that the adhesive completely fills the tortuous crack and forms a continuous adhesive–matrix interface capable of supporting load transfer. Semi-circular bend (SCB) testing demonstrates a substantially higher peak load and increased apparent structural stiffness after repair under the present semi-circular bend configuration, indicating apparent mechanical enhancement beyond simple load-bearing recovery. Digital image correlation (DIC) and fracture morphology show that repair suppresses notch-tip strain localization, reduces the strain concentration factor, shifts the failure-controlling zone away from the original notch tip, and deflects the crack propagation path. Phase-field simulations further show that the post-repair load-bearing capacity is governed by the adhesive–matrix interfacial strength; once this strength approaches or exceeds the tensile strength of the intact PRPC (~8.3 MPa), the repaired crack path is stabilized, enabling peak-load enhancement while suppressing damage localization along the original crack path and shifting failure to adjacent weaker regions. Overall, this work establishes a promising crack repair approach for highly filled PRPCs, while the underlying interface-controlled mechanism provides guidance for adhesive selection and repair design.

## 1. Introduction

Particulate-reinforced polymer composites (PRPCs) represent a class of multiphase functional materials where a polymer matrix forms the continuous phase and rigid particles serve as the dispersed reinforcement [1,2,3,4,5,6,7,8]. The addition of rigid particles not only enhances the stiffness, wear resistance, and thermal stability of the polymer matrix but also lowers material cost, making PRPCs highly attractive for diverse engineering applications [9,10]. Combining the advantages of light weight, tailorable functionality, ease of processing, low cost, and robust environmental adaptability, PRPCs are extensively utilized across aerospace, electronics, civil engineering, and biomedical domains [11,12,13,14,15]. In service, these materials often function as load-bearing structural components and are consequently subjected to complex mechanical and environmental loads. Hence, a thorough understanding of their mechanical behavior and structural integrity is critical for ensuring safe and reliable performance [1,16,17].

The distinctive mechanical behavior of PRPCs arises from their mesoscopic structure [18,19]. A competition between interfacial debonding and matrix shear yielding results in pronounced asymmetry between tension and compression, with the tensile strength being substantially lower than the compressive one. In addition, the polymer matrix exhibits viscoelasticity, and the particle–matrix interface is dynamically active, which together give rise to notable loading rate effects and rheological responses [20,21,22,23]. Owing to the restricted mobility of polymer molecular chains and limited matrix plasticity caused by rigid particles, PRPCs are typically brittle, exhibiting a fracture strain far lower than that of the neat polymer matrix [24]. This brittleness is particularly severe at high filler loadings, where even a modest tensile stress can trigger interfacial cracking and rapid crack propagation. Therefore, PRPCs are prone to cracking when subjected to tension or sustained loading. Once a crack forms, it tends to propagate along the particle–matrix interface, gradually impairing load-bearing capacity and potentially leading to catastrophic failure, thus endangering safety and reliability [25,26,27]. Given the high cost of component replacement, efficient crack repair techniques are economically beneficial and essential for sustainable lifecycle management [28,29].

Among the existing crack repair strategies, self-healing systems can autonomously recover from damage without any external intervention, making them particularly appealing for embedded or hard-to-reach structures [30,31,32,33]. However, such systems usually rely on the integration of microcapsules or vascular networks loaded with healing agents, which inevitably increases both material and manufacturing complexity [32,34]. Another limitation is that the healing agent can be depleted, so the repair efficiency is often restricted to only one or a few damage events [35]. Even when healing does take place, the restored mechanical properties seldom attain the original level. Furthermore, the healing capability degrades significantly after multiple damage cycles, and the restoration of structural stiffness is generally poor [28,29,36,37]. These drawbacks, along with high technical complexity and cost, severely restrict the practical applicability of self-healing systems for load-bearing PRPC components.

By contrast, external intervention relies on manual operation but offers controllable results, higher process maturity, and reduced cost, positioning it as a more practical option for engineering applications [38,39,40,41,42,43]. This approach permits the use of a wide range of commercially available adhesives and can handle various damage sizes and geometries without altering the original composite formulation. Nevertheless, when applied to incipient cracks in highly filled PRPCs, conventional external techniques—such as bonding fully separated fragments or injecting resin at ambient pressure—suffer from critical limitations [38,39]. Adhesive bonding is only applicable after the component has completely fractured into separate pieces; it cannot be used for closed or narrow cracks that have not yet caused full detachment [44,45,46]. Ambient-pressure resin injection relies solely on capillary action to draw the adhesive into the crack [44,45,47]. For cracks narrower than 10 μm in a highly filled composite, capillary forces are limited, leading to impractically slow infiltration rates and often incomplete filling, especially at the crack tip [44,48,49,50,51]. Therefore, a more efficient and reliable infiltration strategy that can actively drive the adhesive through tight, tortuous cracks is urgently required.

Against this background, the present work proposes a pressure-gradient-driven infiltration method that actively drives a low-viscosity epoxy adhesive through narrow and tortuous crack networks within minutes, achieving complete, full-depth filling of sub-10 μm cracks—a task difficult to achieve using conventional capillary injection or simple bonding. This method is applied to a typical PRPC (95 wt.% BaSO_4_/5 wt.% fluororubber), specifically a non-energetic surrogate for polymer-bonded explosive (PBX). Using scanning electron microscopy (SEM), semi-circular bend (SCB) testing, digital image correlation (DIC), and phase-field fracture simulations, we systematically investigate the microstructural filling quality, quasi-static mechanical performance, full-field strain evolution, and crack propagation behavior of the repaired specimens. Special attention is paid to identifying the critical role of adhesive–matrix interfacial strength in regulating strain localization, damage initiation, and crack path selection, as well as establishing the performance threshold that distinguishes simple mechanical recovery from genuine structural enhancement. The findings not only validate a technically feasible and cost-effective repair method for brittle, highly filled PRPCs, but also reveal the interface-dominated micromechanical mechanism governing post-repair performance improvement—a mechanism that can be generalized to guide adhesive selection, repair process optimization, and durability design for a broader class of PRPC systems.

## 2. Materials and Methods

### 2.1. Material and Specimens

In this work, the PRPC was composed of 95 wt.% barium sulfate particles and 5 wt.% fluororubber serving as the binder. This composition is a non-energetic surrogate material for a PBX formulation, designed to mimic the high filler content, particle-matrix adhesion characteristics, and mechanical brittleness of real explosive composites while eliminating safety hazards.

**Figure 1:**
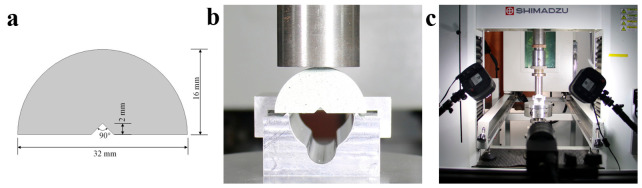
Experimental setup: (**a**) SCB specimen geometry; (**b**) SCB testing configuration; (**c**) DIC measurement system.

The raw materials were mixed and granulated, then hot-pressed in a steel die to form cylindrical blocks. Finally, notched SCB specimens (diameter: 32 mm; thickness: 12 mm; notch length: 2 mm; notch angle: 90°) were machined from the blocks (Figure 1a).

Dumbbell-shaped samples were prepared from the same hot-pressed blocks to determine the intrinsic tensile strength of the PRPC material. Using a SHIMADZU (manufacturer: SHIMADZU Corporation, Kyoto, Japan) universal testing machine, we carried out uniaxial tensile tests at 0.5 mm/min. The results showed a tensile strength of 8.3 MPa and an elastic modulus of 11.2 GPa. These two parameters were adopted as the intrinsic material strength threshold in the phase-field simulations (Section 2.6).

### 2.2. Semi-Circular Bend Test

The specimens were placed on a support fixture featuring two protruding contact points (support span 24 mm) with the notch centered and facing downward; the fixture provided no horizontal constraint, allowing free lateral movement. The loading point was aligned with the notch tip for each specimen. Then, SCB tests were conducted on a universal testing frame under displacement control. A 5 kN load cell was fitted to the frame, and the crosshead speed was kept constant at 0.1 mm/min (Figure 1b). All tests were performed at room temperature (23 ± 2 °C) under ambient humidity, with load-time data acquired at a sampling rate of 20 Hz.

Each specimen was first tested in its undamaged state to obtain the reference load-time curve; the same loading also introduced a well-defined notch-tip crack with a width of less than 10 μm (as confirmed by SEM observation in Section 3). After the crack repair procedure (Section 2.4), the same specimen was retested under identical conditions to evaluate its repaired mechanical response. A total of three specimens were tested. The DIC strain fields presented in the Section 3 are from the second specimen, which is representative of the observed behavior.

### 2.3. Digital Image Correlation

In situ full-field deformation was measured using DIC (Figure 1c). Before bending, a random speckle pattern was generated by spraying a white base coat and then black paint. A Point Grey GRAS-50S5M-C CCD camera (manufacturer: Point Grey Research Inc., Richmond, BC, Canada; 2448 × 2048 pixels) fitted with a 75 mm lens (manufacturer: FUJIFILM Corporation, Tokyo, Japan; model: FUJIFILM HF75SA-1) was used to capture speckle images at a frame rate of 5 fps. Image processing was carried out with DIC software using a 23 × 23 pixel subset, a 5-pixel step size, a Lagrange strain formulation, and a strain window of 5 data points. To minimize out-of-plane displacement, the camera optical axis was aligned perpendicular to the specimen surface, and the specimen was carefully positioned to maintain stable contact with the supports. Loading directions were carefully adjusted to ensure pure in-plane bending of the SCB specimens.

### 2.4. Crack Repair Procedure

The cracks introduced during the initial SCB testing were repaired using a pressure-gradient-driven infiltration method. A low-viscosity epoxy adhesive (Araldite 2020, Huntsman Advanced Materials, Basel, Switzerland) was employed. The manufacturer’s datasheet specifies a tensile strength of 55 MPa, a modulus of 2170 MPa, and a break elongation of 11.9% for the adhesive. Cure schedules are 24 h at room temperature or 16 h at 40 °C (testing temperature 23 °C). At 25 °C, the mixed viscosity falls within 150–300 mPa·s. The pot life (100 g at 23 °C) is roughly 160 min, and the material sets in 16 h at ambient temperature.

**Figure 2:**
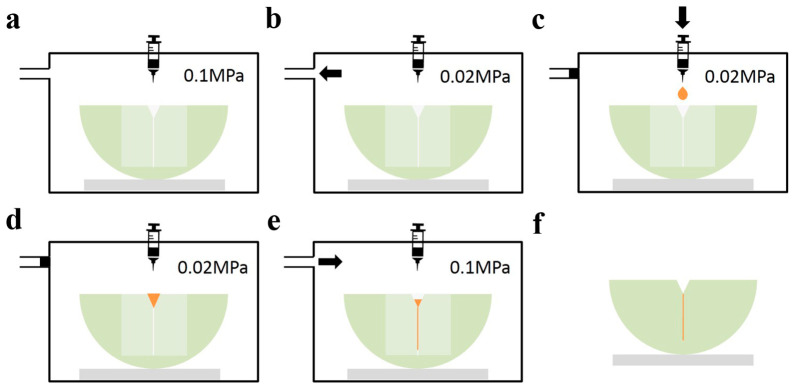
Schematic diagram of the crack repair procedure: (**a**) tape sealing; (**b**) vacuum extraction; (**c**,**d**) adhesive injection; (**e**) vacuum release; (**f**) static curing.

Prior to repair, the adhesive was prepared according to the manufacturer’s instructions, loaded into a syringe, and a locking clip was installed between the plunger flange and plunger rod to prevent accidental depression. The side cracks on the specimen surface were sealed with a polytetrafluoroethylene (PTFE) tape (Chukoh ASF-110FR, 0.08 mm thick, 13 mm wide) to avoid air leakage. The syringe containing the adhesive was inserted into a sealed port on top of the airtight container. The specimen was then placed notch-up inside the same container (Figure 2a). The container was evacuated to a vacuum of 0.02 MPa (Figure 2b). Once the desired vacuum was reached, the locking clip was removed, and the adhesive was injected into the notch (Figure 2c,d). After the vacuum was released to atmospheric pressure, the adhesive fully infiltrated the crack (Figure 2e). Following a dwelling time of 1 min, the specimen was removed from the container. The PTFE tape was then peeled off, and excess adhesive was gently absorbed with a tissue and carefully wiped from the specimen surface using a cotton swab, ensuring no damage to the surface layer. Finally, the specimen was cured at room temperature for 24 h (Figure 2f).

### 2.5. Scanning Electron Microscopy

After repair, the specimens were sputter-coated with platinum to ensure electrical conductivity. A field-emission SEM (10 kV, WD 9.1 mm, SE2) was used to observe the microstructures. Representative images, presented in Figure 4, were acquired at a magnification of 800×. The vacuum level in the specimen chamber was maintained at approximately 1.07 × 10^−3^ Pa during imaging.

### 2.6. Phase-Field Simulations

To investigate the influence of adhesive–matrix interfacial strength on the mechanical response and crack propagation behavior of the repaired PRPC specimens, we performed phase-field simulations. The bulk fracture behavior was described using the viscoelastic phase-field framework proposed by Yuan and Guan [22], including the constitutive relation, strain-energy decomposition, fracture evolution formulation, and numerical solution strategy. A two-dimensional plane-strain SCB model was established with the same specimen geometry, notch position, loading point, and support configuration as those used in the experiments. Displacement-controlled loading was applied.

The bulk fracture was modeled using an AT2-type phase-field formulation with a characteristic length of $l_{c}=0.4\;mm$. For the AT2 model, the tensile strength $\sigma_{t}$, elastic modulus E, critical energy release rate $G_{c}$, and phase-field length scale $l_{c}$ are related by $\sigma_{t}=\sqrt{27EG_{c}/(256l_{c})}$; accordingly, $G_{c}$ was calculated as $G_{c}=256l_{c}\sigma_{t}^{2}/(27E)$ [52]. The matrix strength of the PRPC was obtained from tensile tests of the same batch of material and was approximately 8.3 MPa, while the elastic modulus was measured as 11.2 GPa. To represent the repaired crack interface within the same variational framework, a diffuse interfacial zone was embedded along the original crack path. The width of this diffuse interface does not represent the actual physical thickness of the adhesive–matrix interface, but is a numerical regularization length for interfacial damage evolution. To avoid introducing an additional length parameter, it was taken identical to the phase-field characteristic length ${\;l}_{c}$, consistent with existing diffuse-interface phase-field descriptions of interfacial fracture.

**Figure 3:**
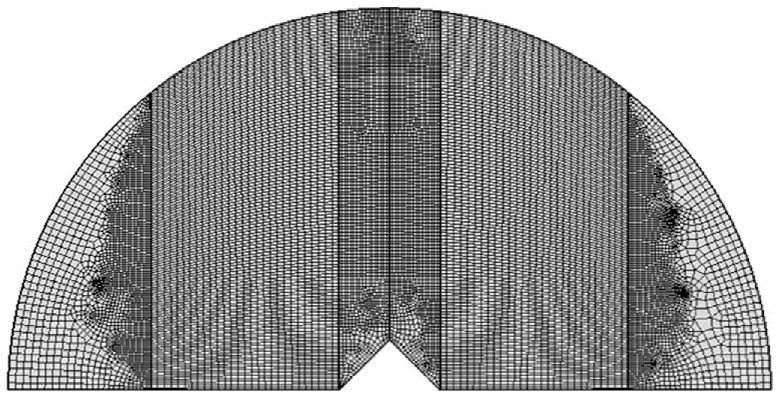
Finite element mesh of the SCB specimen (14,894 elements, 15,073 nodes).

The model was discretized using first-order four-node quadrilateral plane-strain elements. A total of 14,894 elements and 15,073 nodes were generated. Local mesh refinement was applied near the notch and within the key region approximately 10 mm to both sides of the central axis, where the maximum element size was set to $h_{max}=l_{c}/3$ to ensure sufficient resolution of the diffused crack band. The finite element mesh is shown in Figure 3. The coupled displacement and phase-field equations were solved using a staggered scheme. Time discretization was performed using a backward-difference method, automatic time stepping was adopted during loading, and the nonlinear iteration tolerance was set to 0.001.

The adhesive–matrix interfacial strength was systematically varied to evaluate its influence on peak load, damage evolution, and crack propagation path. The intrinsic matrix strength of the PRPC, approximately 8.3 MPa, was used as the reference value for assessing the interfacial strengthening effect. In the damage contour plots, the displayed region corresponds to *d* < 0.99, while regions with *d* ≥ 0.99 were regarded as fully damaged crack regions.

## 3. Results

**Figure 4:**
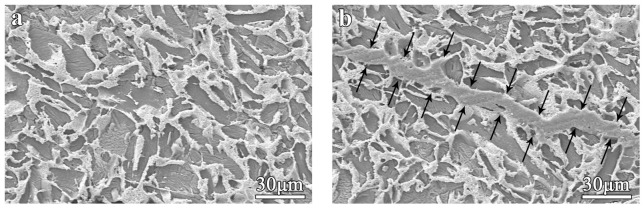
SEM micrographs of a repaired specimen: (**a**) intact region (no crack); (**b**) crack-repaired region (crack fully infiltrated by adhesive, indicated by black arrows).

To evaluate repair efficacy, SEM was performed. Figure 4 presents micrographs of a repaired specimen, showing an intact region (Figure 4a) and a crack-repaired region (Figure 4b). The intact region (Figure 4a) displays the typical PRPC microstructure, with barium sulfate particles (tens of micrometers) randomly embedded in a network-like matrix. In contrast, the crack-repaired region (Figure 4b) exhibits a narrow tortuous crack (width < 10 μm) fully infiltrated by the adhesive, forming a robust interfacial bond. These results confirm that pressure-gradient-driven infiltration effectively fills the crack and restores local material continuity.

**Figure 5:**
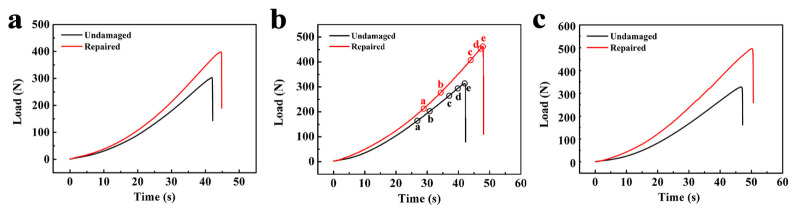
Load-time curves of the three SCB specimens in their undamaged and repaired states: (**a**) Specimen 1; (**b**) Specimen 2; (**c**) Specimen 3.

Figure 5a–c presents the load–time curves of the three SCB specimens in their undamaged and repaired states. All curves exhibit typical brittle fracture behavior: a monotonic increase in load to a peak followed by an abrupt drop, indicating catastrophic failure. Compared with the undamaged curves (black), the repaired curves (red) show steeper initial slopes and higher loads at the same time points. The peak loads of the undamaged specimens are 303.25 N, 313.28 N, and 327.97 N (mean ± SD: 314.8 ± 12.4 N). After repair, the peak loads increase to 397.79 N, 462.53 N, and 495.68 N (mean ± SD: 452.0 ± 48.9 N), corresponding to individual improvements of 31.2%, 47.7%, and 51.1% (average enhancement: 43.6%). These results indicate that the proposed repair method not only fully recovers the load-carrying capability of the cracked specimens but also notably enhances their apparent structural stiffness and load-bearing performance under the SCB configuration.

**Figure 6:**
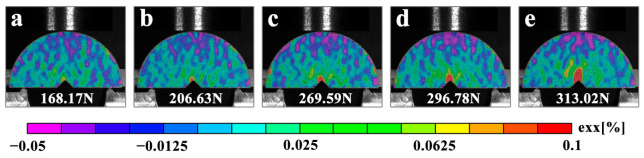
Horizontal strain (ε_xx_) contour plots of Specimen 2 in its undamaged state at discrete loading stages: (**a**) 168.17 N; (**b**) 206.63 N; (**c**) 269.59 N; (**d**) 296.78 N; (**e**) 313.02 N.

**Figure 7:**
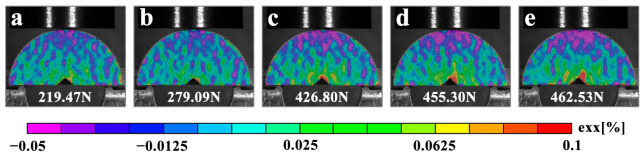
Horizontal strain (ε_xx_) contour plots of Specimen 2 in its repaired state at discrete loading stages: (**a**) 219.47 N; (**b**) 279.09 N; (**c**) 426.80 N; (**d**) 455.30 N; (**e**) 462.53 N.

DIC was employed to quantify the full-field deformation occurring during loading, and here we present the results of Specimen 2 (Figure 5b). Figure 6 and Figure 7 present horizontal strain (ε_xx_) distributions at discrete loading stages (labeled a–e in Figure 5) for the undamaged and repaired states, respectively. In the undamaged state, a localized strain band initiates at the prefabricated notch tip under 150–200 N (Figure 6a). Progressive loading drives continuous propagation upward (Figure 6b–d), leading to fracture upon complete strain localization at peak load (Figure 6e). In the repaired state, localization initiates at 200–250 N, with the strain band originating adjacent to the notch tip in the upper-right region (Figure 7a). The band then propagates laterally with significantly reduced kinetics (Figure 7b–d). Intense localization at peak load indicates crack nucleation at the lateral notch edge (Figure 7e).

**Figure 8:**
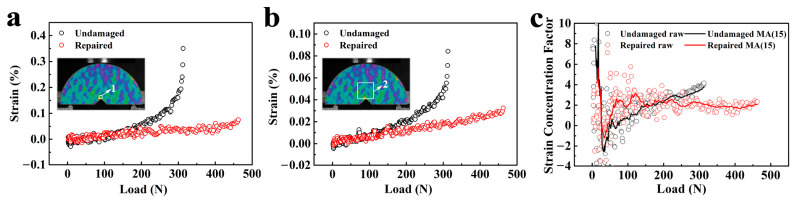
Strain localization analysis: (**a**) strain evolution in ROI 1; (**b**) strain evolution in ROI 2; (**c**) strain concentration factor (SCF = ε_ROI1_/ε_ROI2_).

Figure 8 presents quantitative DIC results to evaluate the effect of crack repair on strain localization. Figure 8a shows the evolution of the average horizontal strain (ε_xx_) within a fixed rectangular region of interest (ROI 1, 0.8 mm × 0.8 mm) at the notch tip, while Figure 8b displays the strain evolution within a larger reference region (ROI 2, 6 mm × 6 mm) enclosing the notch tip. In both ROIs, the undamaged state exhibits a rapid, non-linear increase in strain as the load exceeds 200 N, reaching maximum values of 0.36% (ROI 1) and 0.08% (ROI 2) prior to failure. In contrast, the repaired state shows a significantly suppressed strain response in both ROIs, with a near-linear growth and much lower maximum values (0.07% in ROI 1 and 0.03% in ROI 2), indicating a slower evolution of notch-tip strain localization after repair.

To quantify the severity of strain concentration, Figure 8c plots the strain concentration factor (SCF), defined as the ratio of the average strain in ROI 1 to that in ROI 2 (i.e., SCF = ε_ROI1_/ε_ROI2_). For the undamaged state, the SCF increases sharply beyond 150 N, reaching values of approximately 4 near failure, indicating severe strain localization at the notch tip. For the repaired state, the SCF remains significantly lower (mostly below 2.5) and exhibits a much slower, more stable increase throughout the entire loading history. This quantitative evidence demonstrates that the pressure-gradient-driven repair not only reduces the absolute strain magnitude but also effectively mitigates strain concentration at the notch tip, delaying the onset of local damage and suppressing notch-tip-dominated strain localization.

**Figure 9:**
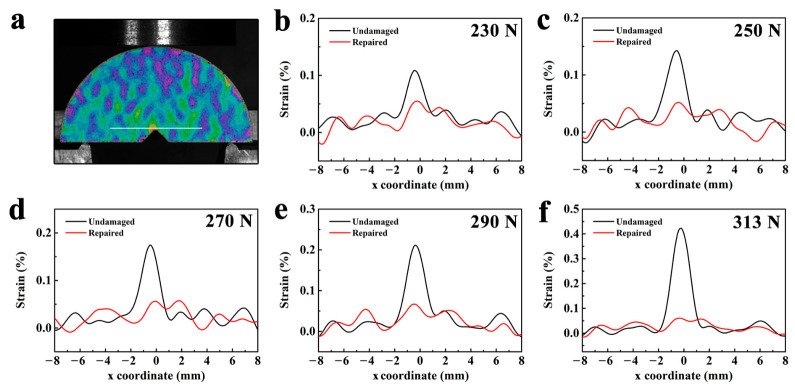
Strain profiles along the extraction line (notch tip at x = 0): (**a**) Extraction path; (**b**–**f**) horizontal strain profiles at ~230–313 N.

To further characterize the spatial distribution of strain localization, Figure 9 presents the horizontal strain (ε_xx_) profiles extracted along a vertical line through the notch tip (x = 0) perpendicular to the strain localization band (Figure 9a), at five load levels: ~230 N, ~250 N, ~270 N, ~290 N, and ~313 N (Figure 9b–f). For the undamaged state, a sharp strain peak develops exactly at the notch tip (x = 0 mm), and the peak value increases rapidly with applied load, rising from 0.11% at 230 N to 0.42% at 313 N. This pronounced and highly concentrated peak indicates severe strain localization at the notch tip. In contrast, for the repaired state, the strain peak is substantially suppressed at all load levels. The maximum strain remains within a narrow range of 0.05–0.07% throughout the entire loading process, with only a minor upward trend as the load increases. Compared to the undamaged state, the peak strain reduction reaches approximately 45% at 230 N and escalates to 83% at 313 N. Furthermore, the repaired curves exhibit a nearly flat profile without a sharp peak, indicating a more uniform strain distribution across the region. These quantitative results complement the ROI-based strain analysis in Figure 8 by directly visualizing the suppression of the strain peak at the notch tip, further confirming that the pressure-gradient-driven repair effectively mitigates strain localization.

**Figure 10:**
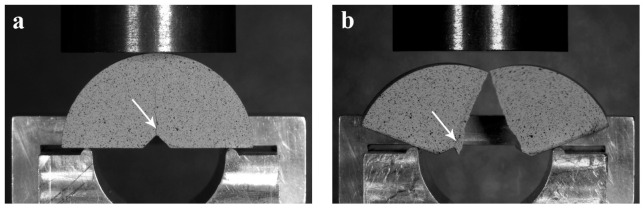
Fracture morphology of the specimen in its (**a**) undamaged and (**b**) repaired states.

Figure 10 displays fracture morphologies of the specimen in its undamaged and repaired states. In the undamaged state, a primary crack (white arrow, Figure 10a) initiates at the notch tip, propagates radially toward the apex, and arrests at ~80% of the specimen height. In the repaired state, crack nucleation occurs laterally adjacent to the notch tip, propagating subparallel to the original crack (white arrow, Figure 10b) toward the apex, ultimately traversing the full height and completely bisecting the specimen.

## 4. Discussion

### 4.1. Crack Filling and Interfacial Strengthening as the Basis of Repair

The SEM observations in Figure 4 directly demonstrate that the pressure-gradient-driven infiltration strategy can effectively drive the adhesive into the narrow (width < 10 μm) and tortuous crack. No obvious voids or unfilled regions are visible in the repaired crack, indicating that the applied pressure gradient overcomes both the capillary resistance and the geometric tortuosity that typically hinder adhesive infiltration in highly filled PRPCs. For successful mechanical repair, complete crack filling is a prerequisite, because any residual unfilled region would act as a traction-free defect, allowing the original crack to reopen during reloading.

More importantly, the repair does not merely fill the crack geometrically. The cured adhesive bridges the two fracture faces and forms adhesive–matrix interfaces along the original crack path, thereby converting the original traction-free crack into a repaired interfacial zone that possesses a finite load-transfer capability. Thus, the repaired crack is no longer an open defect but an adhesive-bridged zone with finite load-transfer capability. This microstructural restoration provides the structural basis for the subsequent alteration in mechanical response. Whether the repaired path remains stable during reloading depends primarily on the adhesive–matrix interfacial strength: a weak interface would promote debonding and reopening along the original crack path, whereas a strong interface can suppress damage evolution along the repaired path and force fracture to occur in adjacent weaker regions.

### 4.2. Suppressed Strain Localization and Shifted Failure-Controlling Zone

Although the repaired state exhibits a higher peak load than the undamaged one (Figure 5), this increase alone does not fully reveal the underlying repair-induced enhancement. DIC analysis (Figure 6, Figure 7 and Figure 8) reveals that the repair fundamentally alters the evolution of local deformation. As shown in Figure 6, the undamaged specimen develops a pronounced ε_XX_ localization zone from the original notch tip as the load increases, with the strain band progressively intensifying and propagating toward the loading point. This indicates that the original notch tip is the dominant strain-localization site in the intact notched specimen. Quantitative DIC analysis (Figure 8) further reveals that both the notch-tip strain and the strain concentration factor rise continuously throughout loading, indicating a damage–localization feedback near the original notch tip: local damage, including matrix microdamage and particle–matrix interfacial debonding, progressively reduces the local effective stiffness and further amplifies the strain concentration. In contrast, the repaired specimen exhibits a much weaker localization process near the original notch tip. During most of the loading process, no sharp strain-localization band develops at the original notch tip (Figure 7). Although the local strain still increases gradually with load, the strain field remains comparatively diffuse, and the main localization region shifts away from the original notch tip. This trend is quantified in Figure 8, where both the notch-tip strain and the strain concentration factor of the repaired specimen remain much lower than those of the undamaged specimen. Notably, this reduced DIC-based strain concentration should not be interpreted as a reduction in the elastic notch concentration factor, nor as a stiffness-enhancement effect of the adhesive. Instead, it indicates that damage evolution along the original notch/crack region is suppressed after repair. The high-strength adhesive–matrix interface restricts reopening and debonding along the repaired crack path, thereby reducing both the degree and the evolution rate of local damage near the original notch tip.

Together, a coherent mechanism emerges. Once damage localization along the original notch/crack region is suppressed by the repaired interface, the original notch tip ceases to be the failure-controlling site. As loading proceeds, the critical deformation zone migrates to neighboring matrix or interface regions where local microstructural defects or weaker particle-matrix interfaces may exist. This is directly supported by the fracture morphologies (Figure 10b), which show a shifted crack initiation site and a deflected propagation path. Hence, the weaker notch-tip strain localization and the altered crack trajectory are not independent phenomena but two manifestations of the same underlying mechanism: the repaired interface suppresses damage localization along the original crack path and transfers failure to a new weaker region.

### 4.3. Interfacial-Strength-Governed Load-Bearing Enhancement and Crack Deflection

**Figure 11:**
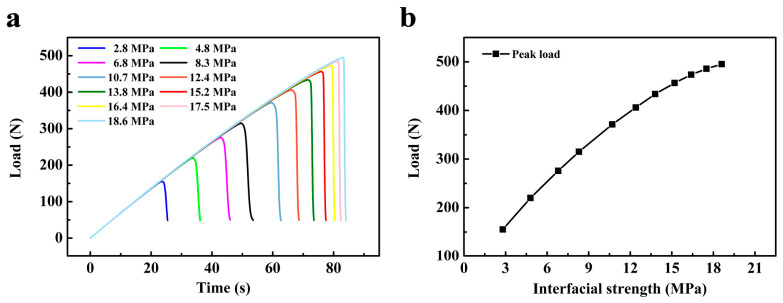
Effect of interfacial strength on mechanical response: (**a**) representative load time curves; (**b**) peak load as a function of interfacial strength.

**Figure 12:**
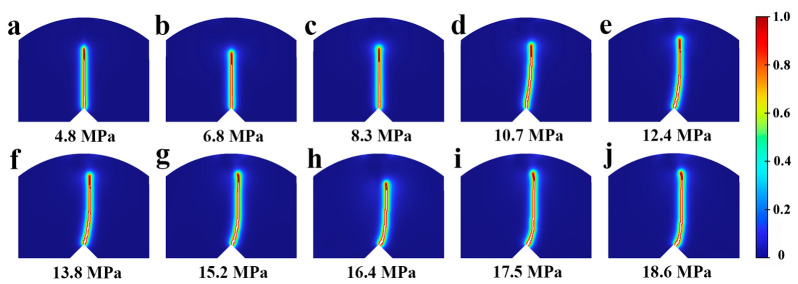
Crack propagation paths at different interfacial strengths: (**a**) 4.8 MPa; (**b**) 6.8 MPa; (**c**) 8.3 MPa; (**d**) 10.7 MPa; (**e**) 12.4 MPa; (**f**) 13.8 MPa; (**g**) 15.2 MPa; (**h**) 16.4 MPa; (**i**) 17.5 MPa; (**j**) 18.6 MPa. The colormap indicates the damage state, and values 0 and 1 correspond to no damage and full damage, respectively.

**Figure 13:**
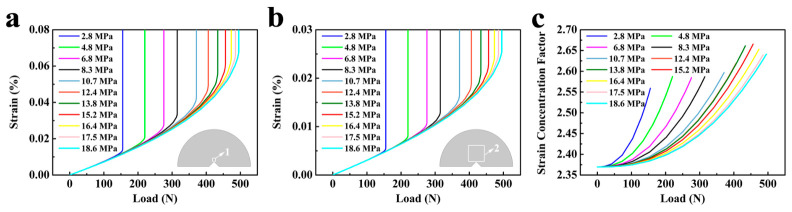
Simulated strain-localization evolution at different interfacial strengths. (**a**) strain evolution in ROI 1; (**b**) strain evolution in ROI 2; (**c**) strain concentration factor (SCF = ε_ROI1_/ε_ROI2_).

**Figure 14:**
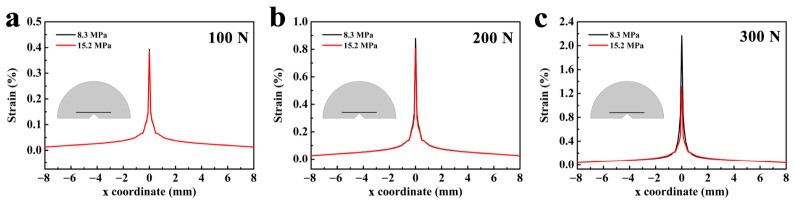
Simulated strain profiles along the extraction line for representative interfacial strengths: (**a**) 100 N; (**b**) 200 N; (**c**) 300 N.

The phase-field simulations further elucidate the critical role of adhesive–matrix interfacial strength. Figure 11, Figure 12 and Figure 13 present a systematic parametric analysis in which the interfacial strength was varied from 2.8 MPa to 18.6 MPa, while Figure 14 provides a direct comparison between representative simulation cases and the experimental DIC results. The simulations address two questions: first, how interfacial strength controls the repaired mechanical response; and second, whether the simulated strain-localization evolution is consistent with the experimental observations.

Figure 11a demonstrates that a higher interfacial strength leads to a gradually increasing load-bearing capacity in the repaired specimen. The peak load summarized in Figure 11b shows a clear transition around the intrinsic matrix strength of approximately 8.3 MPa. When the interfacial strength is lower than this threshold, the repaired interface remains a weak path, and the improvement in peak load is limited. Once the interfacial strength approaches and exceeds the matrix strength, the repaired path becomes sufficiently stable to suppress premature failure along the original crack. The peak load therefore increases markedly and gradually tends toward a plateau at higher interfacial strengths. This indicates that interfacial strength is the governing parameter for converting crack repair from simple recovery to enhanced apparent load-bearing capacity.

The simulated damage contours in Figure 12 further explain the origin of this transition. At low interfacial strengths, damage initiates and propagates along the repaired crack path, indicating that the original crack can still reopen during loading. In this regime, the repaired interface does not fully prevent localization along the original damage path. When the interfacial strength approaches that of the matrix, the crack path begins to deviate from the original notch direction. With further increase in interfacial strength, damage evolution along the repaired crack path is substantially suppressed, and crack propagation shifts into neighboring matrix regions. Therefore, the main function of a strong repaired interface is not to increase the elastic stiffness of the repaired zone, but to maintain interfacial integrity and prevent premature damage localization along the original crack path.

Figure 13 provides additional evidence from the simulated strain-localization evolution. As the interfacial strength increases, both the notch-tip strain and the strain concentration factor decrease significantly at the same load level, and their growth rates during loading are also reduced. This trend is consistent with the DIC observation that the repaired specimen still exhibits gradually increasing strain localization, but its maximum value remains far below that of the undamaged state. Therefore, the reduced strain concentration after repair should be interpreted as suppressed damage-localization evolution around the original notch/crack region, rather than as a reduction in the elastic notch concentration factor.

To directly compare the simulations with the experiments, Figure 14 selects two representative cases. The 8.3 MPa case represents the matrix-strength threshold and reproduces the localization-dominated behavior of the undamaged state, in which strain concentration develops rapidly near the original notch tip. The 15.2 MPa case represents a strengthened repaired interface and reproduces the experimental trend of the repaired state, namely a lower notch-tip strain, a lower strain concentration factor, and a slower localization growth rate. The agreement between Figure 14 and the experimental DIC results in Figure 8 confirms that the proposed phase-field model captures the essential transition from notch-tip-controlled localization to interface-strength-controlled failure migration.

Together, Figure 11, Figure 12, Figure 13 and Figure 14 establish a consistent mechanism. A weak repaired interface allows the original crack path to reopen and limits the load-bearing recovery. A sufficiently strong repaired interface suppresses damage evolution along the original crack path, reduces the growth rate of notch-tip strain localization, and shifts the failure-controlling zone to adjacent weaker regions. This mechanism explains the experimentally observed higher peak load, weaker notch-tip strain concentration, shifted crack initiation site, and deflected crack propagation path after repair. The deflected crack path also implies a longer effective fracture route and additional energy dissipation compared with direct propagation from the original notch tip.

### 4.4. Scope, Transferability and Limitations

Scope. In contrast to the self-healing and conventional external intervention methods discussed in the Introduction, the pressure-gradient-driven infiltration method proposed here does not rely on any pre-embedded system; it is specifically designed for pre-existing, narrow (<10 μm), tortuous, and partially closed cracks that have not yet caused complete fracture, enabling rapid and complete filling. Therefore, this method is not intended to compete with existing approaches; rather, it provides a practical solution for a previously challenging damage state, thereby complementing the current repair toolbox.

Transferability. Although this study focuses on a specific surrogate material, namely 95 wt.% BaSO_4_/5 wt.% fluororubber, the underlying mechanism is expected to be relevant to other highly filled PRPCs in which fracture tends to propagate along weak matrix regions, particle–matrix interfaces, or repaired interfaces. The pressure-gradient-driven infiltration strategy is expected to be effective when the adhesive can sufficiently penetrate the crack and form a repaired interface whose strength is comparable to or higher than the local matrix or particle–matrix interfacial strength. In this sense, the ratio between repaired interfacial strength and matrix strength may serve as a useful criterion for adhesive selection and repair assessment in similar highly filled composites.

Limitations. Several limitations should also be acknowledged. Only one composite formulation and one adhesive were tested, and the repair efficacy was evaluated solely under quasi-static SCB loading, without considering dynamic/cyclic loads, environmental durability, or parametric optimization of the repair process. Quantitative characterization of the adhesive–matrix interface (e.g., energy dispersive X-ray spectroscopy mapping, interfacial fracture energy) was not performed, and the current DIC and phase-field analyses are limited to the specimen scale. In addition, a rigorous fracture-mechanics quantification of crack-path tortuosity, deflection angle, and energy release rate was not performed in the present study. Future work should address these aspects to further validate and extend the proposed repair strategy to other highly filled polymer composites and service conditions.

## 5. Conclusions

This study proposed a pressure-gradient-driven infiltration method to repair narrow (<10 μm) cracks in a highly filled PRPC and systematically examined the repair efficacy together with the underlying mechanisms. The main findings are as follows:

1. Microstructural restoration. The pressure-gradient-driven infiltration enables complete filling of the narrow tortuous crack, forming a dense adhesive–matrix interface that bridges the fracture surfaces and establishes new stress-transfer paths.
2. Significant mechanical enhancement. Compared with the undamaged state, the repaired specimens exhibit a higher peak load and increased apparent structural stiffness under the SCB configuration, indicating mechanical enhancement at the specimen level.
3. Suppressed strain localization and altered failure path. The repair alleviates notch tip strain concentration, reduces the evolution rate of strain localization, shifts the failure controlling zone away from the original notch tip, and deflects the crack propagation path.
4. Governing role of interfacial strength. The improvement in load-bearing capacity after repair does not stem from a simple increase in structural stiffness but is dictated by the adhesive–matrix interface strength, which controls damage suppression and deformation redistribution.

In summary, this work establishes a pressure-gradient-driven infiltration method that enables rapid repair of sub-10 μm cracks in a highly filled PRPC, achieving mechanical restoration and genuine enhancement through interfacial strength-controlled damage suppression. The present results provide a practical pathway for extending the service life of PRPCs and offer guidance for designing durable, repairable composite structures in engineering applications.

## Data Availability

The original contributions presented in this study are included in the article. Further inquiries can be directed to the corresponding author.
